# Sustainability of animal-sourced foods and plant-based alternatives

**DOI:** 10.1073/pnas.2400495121

**Published:** 2024-12-02

**Authors:** Matin Qaim, Rodolphe Barrangou, Pamela C. Ronald

**Affiliations:** ^a^Center for Development Research (ZEF), University of Bonn, Bonn 53113, Germany; ^b^Institute for Food and Resource Economics, University of Bonn, Bonn 53113, Germany; ^c^Department of Food, Bioprocessing and Nutrition Sciences, North Carolina State University, Raleigh, NC 27695-7624; ^d^Department of Plant Pathology and the Genome Center, University of California, Davis, CA 95616-8751

Animal-sourced foods (ASFs)—including meat, dairy, eggs, and fish—are among the most contentious topics in the broader public debates and controversies about sustainable food systems, climate change, biodiversity loss, and healthy nutrition (1–6). This focus on ASFs is unsurprising, as their production and consumption directly relate to numerous key sustainability metrics, both in positive and negative ways.

In terms of positive contributions to sustainable development, ASFs are rich sources of nutrients required for human nutrition, notably proteins, vitamins, and minerals. Hence, ASF consumption can help reduce widespread nutritional deficiencies and thus promote human health (7–10). The production of ASFs also contributes to economic development, as the livestock and fisheries sectors are substantial sources of income and employment for over one billion people worldwide, notably in low- and middle-income countries (11–13). Especially in Africa and Asia, animals are often integrated into the mixed production systems of smallholder farmers (14, 15). Another important role of the livestock sector is that animals—especially ruminants—are able to convert grass and crop residues that are inedible for humans into human food, hence contributing to food and nutrition security on a planet with finite natural resources (1, 16, 17). Similarly, the fisheries sector converts both flora and fauna inedible by humans into human food (13, 18).

In terms of negative contributions to sustainable development, most ASFs have larger environmental and climate footprints than plant-based foods (19). The livestock sector is a major driver of deforestation and global biodiversity loss and accounts for a relatively disproportionate share of agriculture’s greenhouse gas (GHG) emissions (2, 20, 21). Overfishing threatens the world’s oceans and the functioning of marine ecosystems (13, 22). Furthermore, depending on the production systems, livestock husbandry and aquaculture can be associated with animal welfare issues and broader ethical concerns, although improvements have been implemented over time in some systems (23, 24). Finally, intensive livestock production and consumption can be associated with human health issues, increasing the risk of air quality-related health burden, certain chronic diseases, and zoonoses (25–28).

The various negative effects of ASFs are also the main reason why a reduction in global livestock production and consumption is generally considered necessary for enhanced food systems sustainability (1, 3, 29–31). Some studies suggest that at least a 50% reduction in global ASF production and consumption would be required (32), while others calculate that even higher global reductions would be needed, especially for red meat (e.g., beef, veal, lamb, mutton, pork, goat, and venison) to stay within the planetary boundaries (3, 29). Most studies acknowledge that regional differentiation is important because the socioeconomic and environmental conditions can differ remarkably (1). Calls by some that vegetarian or vegan diets for all people should be the goal are not only unrealistic in the short to medium timeline but also tend to neglect or ignore some of the tradeoffs, given that ASFs also have notable positive effects (6–15).

Nevertheless, how to achieve more sustainable patterns of ASF production and consumption and what role plant-based alternatives and other types of technical and social innovations could play are important considerations for research and policy-making and are at the center of this PNAS Special Feature. In this introduction to the Special Feature we provide a broad perspective on the topic, describe the specific contributions of the articles included, and discuss important research and policy implications.

To clarify some of the terminology: As mentioned, the term ASFs—as used here—refers to meat, dairy, eggs, and fish. The term plant-based foods refers to any foods (e.g., fruits, leaves, stems, roots, and seeds) derived from agricultural crops, trees, bushes, or other types of plants. However, the term plant-based alternatives typically does not refer to traditional plant-based foods, but to processed plant-based products that try to mimic and replace meat, dairy, egg, or fish. These meat, dairy, egg, or fish analogs are often produced with multiple ingredients and sophisticated processing technologies (33). Examples include plant-based burger patties, sausages, or plant-based milk and cheese alternatives based on soy, oat, almond, or other ingredients. The term alternative proteins is still broader and to some extent ambiguous. It includes plant-based alternatives but also other alternatives, such as insects, cultured (lab-grown) meat, and protein produced from microorganisms through precision fermentation. We will briefly discuss such non-plant-based alternatives as well, even though these are not the focus of this Special Feature.

## Trends in ASF Production and Consumption

**Fig. 1.**
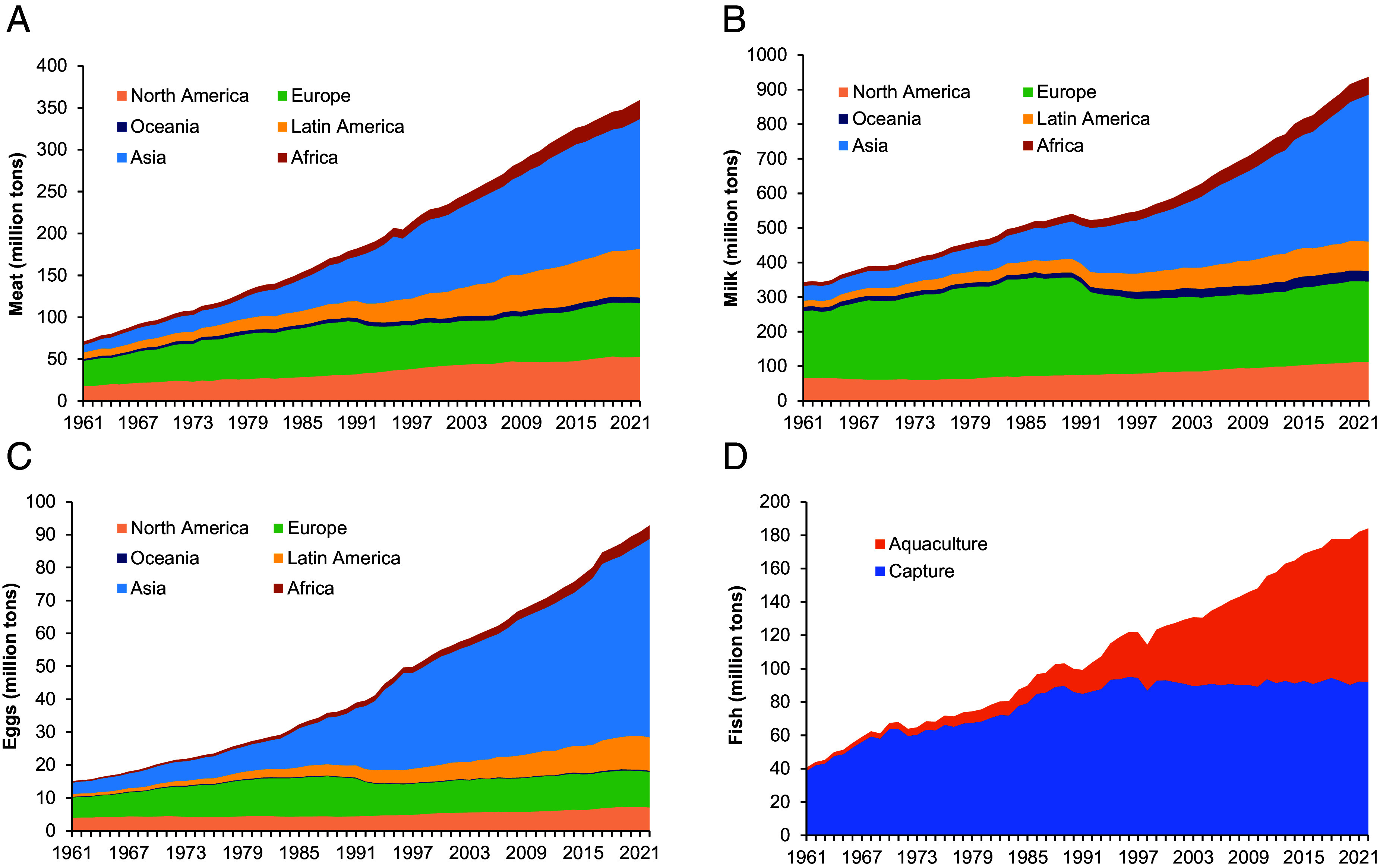
Global production of ASFs (1961–2022). (*A*) Production of meat by region (million tons). (*B*) Production of milk by region (million tons). (*C*) Production of eggs by region (million tons). (*D*) Production of fish, wild capture, and aquaculture (million tons). Figure based on data extracted from refs. 13, 34, and 35.

While certain reductions in the production and consumption of ASFs would be desirable or even necessary for food systems to stay within the planetary boundaries, such reductions are yet to materialize. As a matter of fact, the global production quantities of all types of ASFs continue to increase considerably every year, concurrent with population trends and increases in income, without any sign of slowdown or abatement (Fig. 1). Over the last 60 y, global meat production rose by a factor of five (Fig. 1*A*), global milk production by a factor of almost three (Fig. 1*B*), and global egg production by a factor of six (Fig. 1*C*). Global fish production rose by a factor 4.5 over the last 60 y, even though the quantity of wild capture fish has been stagnating since the 1990s (Fig. 1*D*). The growth since has been entirely driven by aquaculture, including both marine and inland production. For all four types of ASFs, the largest production, and also the largest production growth, is observed in Asia, which is predictable given Asia’s remarkable demographic and economic developments over the last several decades.

**Fig. 2.**
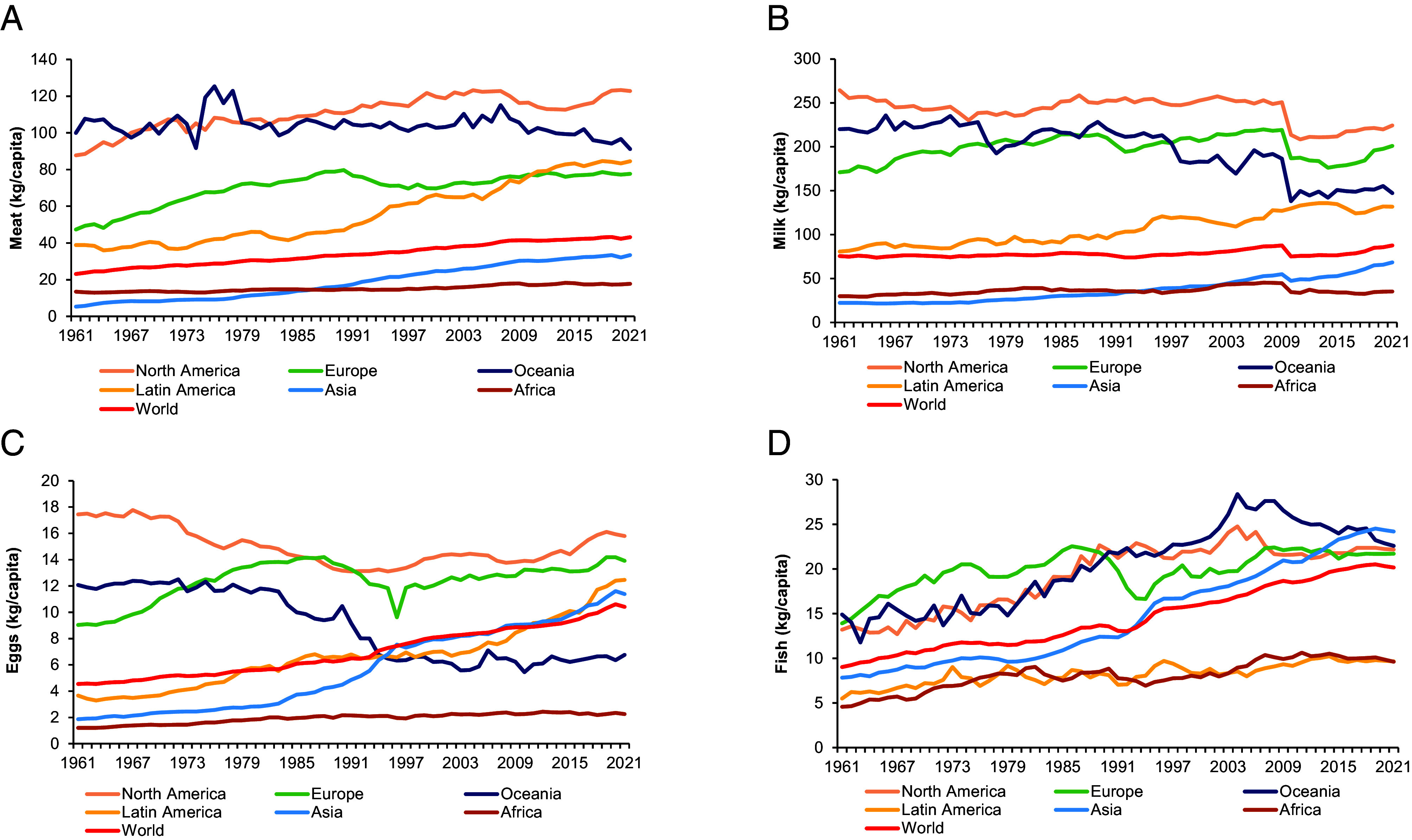
Per-capita consumption of ASFs (1961–2022). (*A*) Annual consumption of meat by region (kg/capita). (*B*) Annual consumption of milk and derived products by region (kg/capita). (*C*) Annual consumption of eggs by region (kg/capita). (*D*) Annual consumption of fish by region (kg/capita). Figure based on data extracted from FAO’s Food Balance Sheets (36). Note that FAO changed some of the details of how the Food Balance Sheets are calculated, which explains the decline observed in 2010 for milk consumption in all regions.

Fig. 2 shows trends and levels of per-capita consumption of ASFs in the various world regions. The differences are substantial, with people in high-income countries consuming significantly more than people in low- and middle-income countries. In 2022, the average consumer in North America consumed around seven times more meat, milk, and eggs than the average consumer in Africa. Poor people tend to eat ASFs relatively rarely and in small(er) quantities: Due to income constraints, their diets are dominated by cereal grains and other plant-based staples as the most affordable and accessible sources of food energy. However, rising incomes lead to more diversified diets and higher ASF consumption, a link that has been consistently observed in all world regions.

North America and Europe seem to have reached peak meat ingestion, with mean per-capita consumption now stagnating at high levels (Fig. 2*A*). For milk and eggs, mean per-capita consumption in North America and Europe has still increased over the last 10 y (Fig. 2 *B* and *C*). This is interesting, because in some high-income communities, vegetarian and vegan lifestyles are increasingly popular. While this may be true in certain population segments, it is not so for the majority of the population and not yet clearly reflected in aggregate consumption statistics. Only in Oceania, per-capita consumption levels have declined for most ASFs.

In the low- and middle-income countries of Africa, Asia, and Latin America, per-capita consumption levels continue to rise, as does the world average, underlining that—in addition to population growth—income growth is another key reason for the continued growth in global ASF production and consumption. It is not clear how this global trend could be reversed any time soon.

## Nutritional Effects of ASFs

Despite the aforementioned trends, malnutrition remains a major global concern. Around 148 million children suffer from stunted growth, over 700 million people are undernourished in terms of food energy, and around 3 billion cannot afford healthy diets that meet all nutritional requirements (37). Poor people and households in low- and middle-income countries are particularly affected by nutrient deficiencies. This situation will likely get worse with climate change, unless major adaptation and mitigation strategies are prioritized.

ASFs contain essential macro- and micronutrients and therefore contribute to reducing nutritional deficiencies. A few micronutrients—such as vitamin B12—are unique to ASFs. Others are contained in higher quantities or in more bioavailable form in ASFs than in plant-based foods, including iron, zinc, calcium, vitamin A, vitamin D, and omega-3 fatty acids (6). Studies with data from various low- and middle-income countries reveal that the consumption of ASFs is positively associated with nutrition and health outcomes (7–10, 38).

In their article in this *Special Feature*, Beal et al. (39) emphasize the role that ASFs can play for ensuring adequate nutrition for people over different life stages. The analysis starts with a definition of the nutritional needs across each stage of the life cycle. Due to their high micronutrient needs, infants and young children, women of reproductive age, pregnant and lactating women, and older adults, especially older women, are identified as nutritionally vulnerable. The roles of different types of ASFs and micronutrient-dense plant-based foods for meeting essential nutrient needs across the vulnerable stages of the life cycle are examined. ASFs and traditional nutritious plant-based foods are shown to be highly complementary in meeting nutritional needs. While plant-based foods can provide most of the essential nutrients in theory, adding even small quantities of ASFs can be advantageous, especially for the nutritionally vulnerable. Iron, zinc, calcium, and vitamin B12 are shown to be critical nutrient gaps related to low ASF consumption. Socioeconomic factors contributing to nutritional vulnerability and broader challenges for achieving the nutrition goals are also discussed.

In their article in this *Special Feature*, Khonje and Qaim (40) analyze the role of ASFs for child nutrition in Africa, where mean ASF consumption is relatively low. Using large microlevel longitudinal datasets from five countries in sub-Saharan Africa (Ethiopia, Malawi, Nigeria, Tanzania, Uganda), they show that ASF consumption significantly increases child linear growth and critically reduces the likelihood of childhood stunting, also after controlling for household wealth and other confounding factors. Within the group of ASFs, eggs and dairy seem to have the most favorable effects. The consumption of nutritious plant-based foods—such as legumes, vegetables, and fruits—is also nutritionally beneficial, but the child anthropometric outcomes are better with additional ASF consumption than without, underlining the nutritional complementarities. These findings do not imply that ASF consumption should not be reduced where consumption quantities are high, but they clearly suggest that improving access to ASFs for poor households in Africa could substantially help reduce child undernutrition. Other studies suggest that this is also true for poor households in Asia and elsewhere (38).

## Environmental Effects of ASFs

ASFs have much larger environmental impacts than plant-based foods. While livestock production accounts for less than one-fifth of the total food energy output, it uses 70% of all agricultural land, and 40% of the arable cropland (2, 16). Also, livestock production accounts for a disproportionately large share of global freshwater use and up to two-thirds of all food-related GHG emissions (21, 30). Livestock production is also considered one of the main drivers of global deforestation and biodiversity loss (1, 20).

**Fig. 3.**
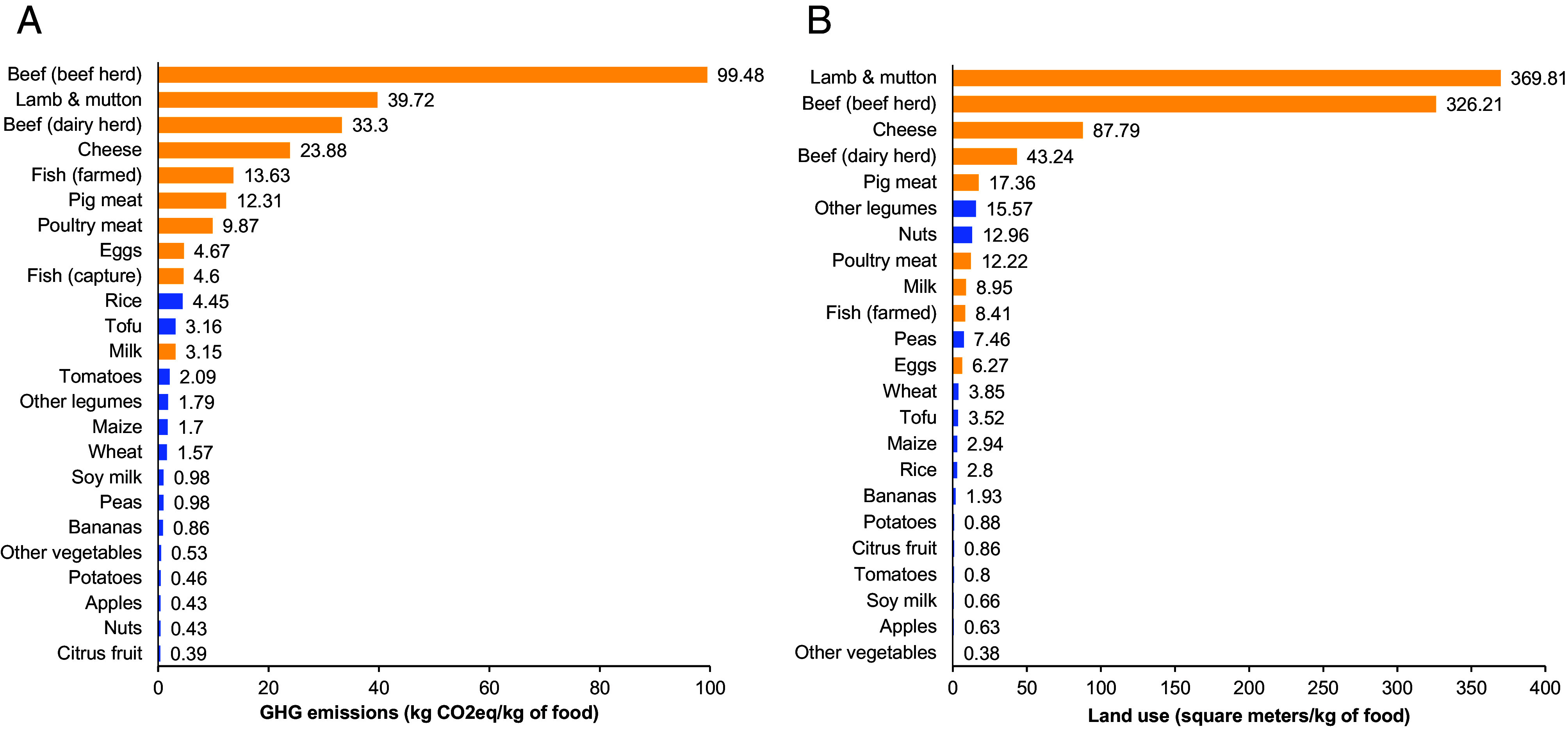
Environmental footprints of animal-sourced and plant-based foods. (*A*) Climate footprints measured in terms of GHG emissions (kg CO_2_ equivalents) per kg of food product. (*B*) Land use footprints measured in terms of square meters of agricultural land per kg of food product. ASFs are shown in yellow, plant-based foods are shown in blue. Figure based on data extracted from refs. 19 and 41.

Fig. 3 shows the environmental footprints of ASFs and selected plant-based foods in terms of GHG emissions and land use per kg of food product. These data represent average results from a meta-analysis covering data from 570 life cycle assessments (LCAs) carried out in different parts of the world (19). The environmental footprints can differ considerably depending on the production technology used, as will be discussed in more detail below. The mean results confirm that ASFs have much larger climate footprints than almost all plant-based foods, even though with notable differences also within the group of ASFs (Fig. 3*A*). Meat from ruminants (beef, lamb, and mutton) is notably performing poorly because ruminants emit a substantial amount of methane, a very potent GHG. The correct calculation of the global warming potential of methane has been debated recently (1, 42). However, while the calculation method has some influence on the quantitative outcomes, it does not change the general finding that ruminant meat has disproportionate climate impacts relative to other types of foods (1).

ASFs also have larger land-use footprints than most plant-based foods, again with meat from ruminants having the most devastating impact (Fig. 3*B*). It is important to note that some of this land used by ruminants is grazing land that could not be used otherwise for food production. Nevertheless, negative effects on biodiversity and soils are widespread in heavily grazed regions (1, 20, 43). In addition, especially in some countries of the Global South, grazing areas are increasingly expanded into forestlands (44).

Another point worth mentioning is that environmental footprints cannot only be expressed per kg of food product, as shown in Fig. 3, but also per unit of protein or other nutrients, which may be useful to better account for the nutritional values of ASFs. However, the relative ranking of foods in terms of their environmental footprints is often unaffected by choosing alternative reference systems (1, 19, 41).

## Role of Production Technology

What the mean environmental footprints shown in Fig. 3 conceal is that the environmental effects crucially depend on the production technology. For instance, the climate and land-use footprints of beef producers in the highest and lowest percentile worldwide can differ by a factor of >10 (19). This is perhaps unsurprising given that cattle in Africa is held and managed under very different conditions than cattle in Europe or North America.

In her perspective article in this *Special Feature*, Van Eenennaam (45) reports that the larger part of the global livestock-related GHG emissions occurs in low- and middle-income countries, mainly because of the lower relative productivity there, which results from traditional technologies and husbandry systems. In contrast, in high-income countries, more efficient systems have been developed that reduce the environmental footprint per unit of output. Given that the demand for ASFs in low- and middle-income countries will likely increase the most, the adoption of technologies to improve production efficiency there is seen as key to both reducing livestock emissions and providing affordable access to ASFs to improve human nutrition. Concrete examples from India, Brazil, and other regions are discussed, illustrating how strategic opportunities in geopolitically important countries are shaping and evolving.

Promising technologies in the livestock and fisheries sectors include improvements in animal management and health, genetics, and feeding practices, among others. Animal management and health involve aspects such as herd organization, grazing and manure management, and veterinary services that help to improve animal productivity and reduce morbidity and mortality (46, 47). Genetics involves the development and use of improved animal breeds. New genomic approaches can help to increase the precision and speed of developing desirable animal traits related to productivity, fecundity, disease resistance, etc. (48). Improved animal feeding practices include the use of forages with higher digestibility, as well as feed additives to mitigate enteric methane emissions in ruminant livestock (46). The use of circular feeds in livestock and fish production also has potential to reduce the environmental footprint of ASFs significantly (17, 18, 49). Circular feeds are low-opportunity-cost feeds—such as crop and food by-products, residues, and wastes—that cannot be eaten by humans directly. In some cases, prior use of fermentation processes or insect larvae can help to extract nutrients from manure and other wastes and thus upcycle valuable resources for circular food systems and bioeconomies (49, 50).

In their article in this *Special Feature*, Meo-Filho et al. (51) analyze the effects of one concrete feeding technology on reducing GHG emissions from grazing cattle. Specifically, they carried out an experiment testing the effectiveness of a seaweed-based feed additive as an enteric methane inhibitor in grazing beef cattle, comparing the treatment group to a control group that received no feed additive. No differences in weight gain were observed between the groups, but average enteric methane emissions were reduced by more than one-third using this treatment. These results suggest that improved technologies could play an important role in reducing the climate footprint of ASFs.

Convincing livestock and aquaculture farmers to use new types of environmentally friendly inputs and technologies is easier when these innovations also help to increase productivity and profits but may be more challenging when the private benefits for farmers are small. Researchers have not yet determined the extent to which farmers are willing and able to adopt technologies that reduce environmental impacts and thus benefit society but without any gains for farmers themselves, either in terms of reduced costs or higher profits. The successful implementation of such technologies will depend on governments providing the right incentive system through fiscal or regulatory policies. In addition, private environmental standards set by large companies or industry associations can potentially play an important role.

Stringent public or private environmental standards are conceivable in high-income countries, where the negative environmental impacts of farming are often prioritized by policy-makers and civil society. In low- and middle-income countries, the priorities may be different, so technologies that reduce environmental impacts through higher productivity may be easier to implement. Recent research shows that livestock production increases and simultaneous reductions in GHG emissions do not always require new technologies but may also be possible with existing technologies by reducing prevalent inefficiencies in input use (52).

More generally, there is widespread consensus that agricultural technologies and improved animal husbandry systems have an important role to play for making food systems more sustainable, but in addition to, not instead of, changing diets and consumption patterns.

## Plant-Based Alternatives to ASFs

Most nutrients and beneficial food compounds that the human body needs can be obtained from traditional plant-based foods, meaning that large quantities of ASFs are not needed physiologically. For instance, legumes are particularly rich in protein, whereas vegetables, fruits, nuts, and seeds are good sources of micronutrients and essential fatty-acids (6, 39). This is why people eating no or low quantities of ASFs are especially advised to consume a variety of plant-based food groups in balanced proportions. However, as mentioned, the term plant-based alternatives mostly refers to processed plant-based foods that try to mimic meat, dairy, egg, or fish products. These meat, dairy, egg, or fish analogs are typically produced by food companies, targeting consumers who prefer “real” ASFs but are willing to reduce consumption for ethical, environmental, or other reasons, yet without wanting to radically change the types of meals consumed (53–55).

Mimicking the taste and texture of whole cuts of meat or fish is still technically difficult with existing technologies, so plant-based meat or fish analogs come mostly in the form of burgers (patties), nuggets, sausages, or crumbles (33). For plant-based milk alternatives, various types—such as soy milk, oat milk, or almond milk—have gained some popularity. Food industry interest in plant-based meat and dairy alternatives already started in the late-1990s and early-2000s, but investments were scaled up especially during the last 10 y (56). The number of new manufacturers and brands in these sectors has skyrocketed since 2014 (33). This is also reflected in high market growth rates of plant-based meat and milk analogs between 2014 and 2020, even though the growth has declined more recently in some market segments.

To provide some perspective: In 2020, 13 million tons of alternative protein products were consumed globally (57), which is less than 1% of the more than 1,500 million tons of combined global output of meat, milk, eggs, and fish (Fig. 1). Nonetheless, further growth of plant-based alternatives is projected. Important to note is that so far developments are primarily concentrated in high-income countries. According to a company database (58), 81% of the currently existing alternative protein companies worldwide are located in high-income countries, 19% in middle-income countries (mostly upper middle-income), and none in low-income countries (45). This is perhaps not surprising given that plant-based analogs are significantly more expensive than the ASFs that they try to mimic (33, 45). For lower-income consumers, such plant-based alternatives are currently unaffordable. Bringing down costs will be a major challenge for continued industry growth. Further improving taste, texture, and nutrient profiles will be other challenges to convince more consumers that these are good alternatives for ASFs (53).

In their article in this *Special Feature*, Etzbach et al. (59) evaluate the potentials and constraints associated with utilizing plant proteins from a food technology perspective. While the processing of animal proteins for food production is very well understood, exploration of the functionality of plant-based proteins, with the exception of gluten and soy, is still in its early stages. For this reason, the authors comprehensively evaluate the nutritional profiles and functional characteristics of 61 commercially available protein ingredients from oilseeds, cereals, potatoes, and grain legumes, categorizing them based on their protein content into protein-rich flours (protein content less than 50%), protein concentrates (protein content between 50% and 80%), and protein isolates (protein content higher than 80%). The analysis reveals that the functionality (e.g., emulsifying, solubility, color, particle size, foaming) varies significantly not only between the different ingredients but also between different batches of the same ingredient. The findings demonstrate some of the unique challenges faced when analyzing the quality and usefulness of plant proteins.

## Sustainability Effects of Plant-Based Alternatives

In order to better understand the potentials and constraints of plant-based alternatives, knowledge on how they may affect various sustainability dimensions is important. In his article in this *Special Feature*, Springmann (60) conducts a multicriteria assessment of 24 meat and milk alternatives that integrates nutritional, health, environmental, and cost analyses. The plant-based alternatives include unprocessed plant-based foods, such as beans and peas, traditional meat alternatives, such as tofu, as well as new and highly processed meat and milk alternatives, such veggie burgers, sausages, and different types of plant-based milk. The analysis combines results from existing environmental LCAs with data on nutrient composition, dietary risk factors, and market prices. The calculations show that unprocessed plant-based foods perform best in terms of all criteria. Traditional and new processed plant-based alternatives perform somewhat worse, but still better than ASFs in terms of most criteria. These findings suggest that replacing ASFs with plant-based foods and plant-based alternatives can result in multiple-win situations, at least in high-income countries to which this analysis refers. More generally, plant-based alternatives may help to reduce the projected future growth in ASF demand at the global level.

In their perspective article in this *Special Feature*, Aimutis and Shirwaiker (61) discuss market trends for plant-based meat and milk alternatives and analyze shortcomings of existing environmental LCAs for these alternatives. For example, many of the published plant-based food assessment studies neglect processing impacts in their analyses, which include protein extraction, concentration, isolation, and drying. Furthermore, many environmental LCAs ignore the extensive stainless-steel usage required for processing. Substantial amounts of energy and various types of chemicals are used for protein extraction and concentration processes and for sanitation and equipment sterilization. Ignoring these aspects, as is often done in food-based LCAs, may result in underestimated environmental footprints of plant-based alternatives. Better methods and more comprehensive and reliable metrics are needed to identify and address major constraints for further industry growth.

## Understanding and Changing Consumer Behavior

Reducing ASF consumption and a stronger shift to plant-based foods or plant-based alternatives will also require behavioral change among consumers. Hence, understanding consumer choices and how they can be influenced is important for designing suitable policies (62). In their article in this *Special Feature,* Jahn et al. (63) analyze which types of plant-based alternatives resonate most with consumers and how pricing dynamics influence demand. They conducted studies in the United States with four different burger types, one from beef and the others from different plant-based sources, to evaluate preferences and preference distributions for specific product characteristics. In addition, in an experimental setup, they tested the effects of price changes on product choices. While the beef burger is preferred by most, interesting preference heterogeneities are identified. For example, the authors find significant gender differences between a meat analog (plant-based burger) and semianalog (veggie burger): females preferred the veggie burger, and males preferred the plant-based burger that tastes more like meat. Price changes for the beef burger have smaller effects on demand than price changes for the plant-based alternatives, suggesting that the consumption of plant-based alternatives could increase over-proportionally if costs and prices were further reduced.

In their article in this *Special Feature*, Katare and Zhao (64) carry out an experiment with US consumers in an online grocery shopping environment, testing the effects of two behavioral interventions on consumer choices for plant-based foods and plant-based alternatives. The first intervention is a carbon footprint label. The second intervention involves product categorization, meaning that consumers are presented with a curated category of plant-based alternatives. The data show that carbon footprint labels increase the selection of plant-based alternatives by 37% and categorization by 25%. A combination of both interventions does not further increase the choices for plant-based alternatives. These results suggest that simple low-cost interventions at the online retail level can effectively motivate environmentally friendly food choices for some consumers. However, at the global level, online grocery sales only account for a small proportion of total food retailing at this time. Whether the same results also apply in physical market environments may need further analysis.

Beyond labels and other types of information-related interventions, governments could incentivize more sustainable food choices through taxes and subsidies. However, taxes on meat and other ASFs are unpopular and therefore hardly observed anywhere in the world up till now.

## Diet and Food Systems Modeling

In addition to looking at individual food choices and diets for meeting nutritional needs and preferences, several recent studies have tried to model the implications of global dietary choices and shifts for food systems sustainability (3, 29–32). Such global modeling exercises necessarily have to build on many simplified assumptions, as detailed microlevel data on all links in specific regional settings are not available. A landmark study in this direction was the one published in 2019 by the EAT-Lancet Commission (3), in which an interdisciplinary team of researchers developed dietary guidelines to align human health and environmental health objectives for a global population of 10 billion people. These global dietary guidelines also became known as the Planetary Health Diet (PHD). The PHD directly relates to the topic of ASFs and plant-based alternatives, as the PHD recommendations for all ASFs are far below what is currently consumed in most high-income countries. The EAT-Lancet study has led to substantial follow-up work to further improve the data and/or analyze broader implications not covered in the original work.

In their article in this *Special Feature*, Gu et al. (65) provide estimates of international adherence to the PHD using the Planetary Health Dietary Index (PHDI). In addition, data on links between diets and mortality from three long-term cohorts of adults in the United States are used to estimate how many cases of mortality could be prevented through shifts from current national diets to the reference PHD. Results suggest that a large number of deaths could be prevented, especially deaths related to cardiovascular, respiratory, and infectious diseases, and cancer, all of which are often associated with unhealthy diets. While the analysis covers all world regions, the results are probably most reliable for high-income countries given that the cohort studies used for the modeling analysis are all from the United States. Further research with more detailed nutrition and health data from low- and middle-income countries will be useful, once such data are becoming available.

In their article in this *Special Feature*, White and Hall (66) employ a different approach to model food systems. They use multiple-source data for 153 countries to analyze the “optimal” role of ASFs in national food systems. The data used include agricultural production and trade data for a large number of commodities, food security indicators, nutrition and health data, food price data, GHG emissions, demographic data, and macroeconomic indicators, among others. The country-level data are correlated and used to generate Bayesian Learning Networks, assessing directional associations. The results are also used to support a simulation exercise to evaluate the optimal role of ASFs under local/regional food system conditions and specifically defined sustainability goals. Depending on local conditions and goal specification (e.g., priority for climate versus nutrition goals), the optimal role of ASFs differs drastically. However, all simulated optimal scenarios include ASFs. Remarkably, a major conclusion of the article is that approximately 15% of overall food supply should actually originate from ASFs, with the noteworthy accompanying finding that emissions are not systematically minimized when ASFs are fully excluded. This is a reminder that mixed and balanced food systems should be considered and that strict elimination of ASFs is arguably not supported, nor optimal.

## Insects, Cultured Meat, and Precision Fermentation

Beyond plant-based alternatives to ASFs, the broader topic of “alternative proteins” also encompasses insect proteins, cultured (or lab-grown) meat, as well as proteins produced by microorganisms in precision fermenters. These technologies are not covered by articles in this *Special Feature*, mainly for reasons of space limitations. All three technologies may have intriguing potential to contribute to protein supplies in the future, but each of them still has its own challenges to deal with. Insects as food have been tried for long but have not gained significant market shares up till now, mainly to due consumer acceptance issues (54). The technologies for cultured meat are not yet fully matured; even though the first products are now appearing on the market in selected countries, they are very expensive and still far away from being a real competition for traditional meat (1, 56, 67). The same is true for precision fermentation. In the long-term, precision fermentation could revolutionize food systems, but the technologies are still in their infancies. Many of the interesting new developments may depend on genetically engineered microorganisms, which could be associated with issues of consumer acceptance in some parts of the world. While these types of food technologies may gain importance and play interesting roles in certain market segments, it is unlikely that they will disrupt global ASF production and consumption in the foreseeable future.

## Conclusion

The articles in this *Special Feature* contribute to a dynamic and timely research field that is core to the evolution and transformation of food systems to enhance human health and environmental health. ASFs lead to important tradeoffs that need to be managed through a mixture of appropriate innovations, smart policies, efficient institutions, and behavioral change. There are no universally applicable blueprints. Rather, diverse transformation paths need to be customized to local conditions and constraints. In most low-income and many middle-income countries, the production and consumption of ASFs will likely continue to grow. This is mostly good from a nutrition and human health perspective, but comes with some environmental challenges. The adoption of improved technologies and grazing management strategies needs to be prioritized to reduce the environmental footprint of ASFs.

In high-income and many upper middle-income countries, mean ASF consumption should be reduced. Highly processed plant-based alternatives can support this transition to some extent, but they are still relatively expensive and need further technological and organoleptic improvements. In any case, consuming more traditional nutritious plant-based foods—such as legumes, vegetables, and fruits—is even more healthy and sustainable and should be the default option for substituting ASFs. Overall, flexitarian diets with low to moderate quantities of dairy and eggs and occasional consumption of meat and fish seem to be compatible with sustainable development.

Further research is needed in this important field, including questions such as the following: How can metrics of food systems sustainability be improved for meaningful international comparison, covering the heterogenous conditions and various sustainability dimensions? How can technologies to improve livestock productivity and reduce environmental externalities be implemented at scale, especially among smallholder farmers in low- and middle-income countries, but also among ranchers and livestock keepers in high-income countries? How can the supply of healthy and nutritious foods affordable to all be improved, in spite of the challenges posed by climate change? Through what interventions can major shifts toward more plant-based diets be achieved? How can efficient supply chains for alternative proteins be developed? This *Special Feature* will hopefully draw attention to this important field and stimulate interdisciplinary follow-up work on these and other sustainable food systems–related questions.
